# Stable functional compensation within hippocampal-subregion networks in patients with temporal glioma before and after surgery

**DOI:** 10.3389/fnins.2022.991406

**Published:** 2022-09-01

**Authors:** Yuhai Zhang, Honghao Xu, Yong Liu, Kun Yang, Yuanjie Zou, Hongyi Liu

**Affiliations:** ^1^Department of Neurosurgery, The Affiliated Brain Hospital of Nanjing Medical University, Nanjing, China; ^2^Department of Functional Neurosurgery, The Affiliated Brain Hospital of Nanjing Medical University, Nanjing, China

**Keywords:** temporal glioma, hippocampal-subregion, functional connectivity, pattern classification, functional compensation

## Abstract

**Objective:**

To identify whether tumor invasion of the temporal lobe induces functional compensation of the hippocampal-subregion (HIPsub) network connectivity before surgery, and to further validate the stability of this functional compensation within the HIPsub network in patients with temporal glioma tumor (TTumor) after surgical resection of the tumor.

**Methods:**

In the first cohort, analysis of HIPsub functional connectivity (FC) was conducted to identify the functional compensation of the altered HIPsub connectivity pattern in TTumor through a pattern classification approach. Then, the second cohort investigated whether functional compensation in TTumor patients changed after surgical resection of the tumor.

**Results:**

In the first cohort, this study identified altered HIPsub network connectivity patterns and its functional compensation regions (i.e., left parahippocampal gyrus and bilateral cerebellum anterior lobe) in TTumor patients. Second, the altered HIPsub network connectivity patterns had the power to discriminate TTumor patients from healthy controls (CN) on an individual subject basis, with an AUC of 97.0%, sensitivity of 93.5%, and specificity of 90.3%. Finally, in the second cohort, we found that functional connectivities of functional compensation regions within the HIPsub network in TTumor patients did not change between before and after surgery.

**Conclusion:**

This study provides novel evidence regarding functional compensation within the HIPsub network in TTumor patients. It has been suggested that the fine hippocampal subregion was more sensitive, which reveals functional compensation induced by tumor invasion of the temporal lobe. Furthermore, this study verified the stability and persistence of this functional compensation in TTumor patients after surgical resection of the tumor.

## Introduction

Gliomas account for approximately 81% of malignant primary brain tumors (Ostrom et al., 2014), with the second-highest proportion of temporal lobe invasion (Ostrom et al., 2019). Damage to the temporal lobe, known to be located in the hippocampal memory network, can alter its function including memory processing, emotional processing, and sensory processing (Chen et al., 2016, 2020, 2022). Interestingly, clinical observations have discovered that memory function remains intact among patients with temporal glioma. In recent years, a large number of studies have demonstrated that local tumor invasion could brain neural remodeling in the form of a network in order to maintain normal cognitive function (Liu et al., 2019, 2020b). However, little is known about whether tumor invasion of the temporal lobe can induce functional compensation of the hippocampal network connectivity. In particular, it is unknown whether this functional compensation can be retained after surgical resection of the tumor. Therefore, it is critical to understand the stability of the functional compensatory mechanism, which facilitates the preoperative planning of the compensatory mechanism of the protective function.

In recent years, neuroimaging studies have suggested functional heterogeneity and different FC patterns within the hippocampal subregion (Davachi, 2006; Robinson et al., 2015; Chen et al., 2016, 2020, 2022). Neuroimaging studies indicated that the hippocampus could be separated into three functional subregions: the anterior emotional region (HIPe), the middle cognitive region (HIPc), and the posterior perceptual region (HIPp) (Robinson et al., 2015; Chen et al., 2020, 2022). Different hippocampal subregions are involved in different cognitive processing (Robinson et al., 2015; Chen et al., 2016, 2020, 2022; Bai et al., 2019). Therefore, it is reasonable to speculate that tumor invasion of the temporal lobe can have different effects on different hippocampal subnetworks. In particular, the hippocampal subnetwork can more accurately describe the compensatory mechanisms that are induced by tumors.

In recent years, a large number of studies have consistently demonstrated that tumor invasion of the brain or lesion of a certain brain region within the network induces different forms of network compensation, and tumors in the different brain regions induce various forms of compensation (Bartolomei et al., 2006; He et al., 2007; Guggisberg et al., 2008; Alstott et al., 2009; Pravata et al., 2011; Liu et al., 2019, 2020b; Yuan et al., 2019). Based on the fact that the temporal lobe is located within the hippocampus network, we hypothesized that tumor invasion of the temporal lobe induces functional compensation of the hippocampal subnetwork. However, whether compensation induced by tumor invasion of the brain can be eliminated by tumor resection is unknown. In order to address these issues, a preoperative and postoperative study design was utilized to identify the stability of this functional compensation in patients with temporal glioma tumor (TTumor) after undergoing surgical resection of the tumor.

**FIGURE 1:**
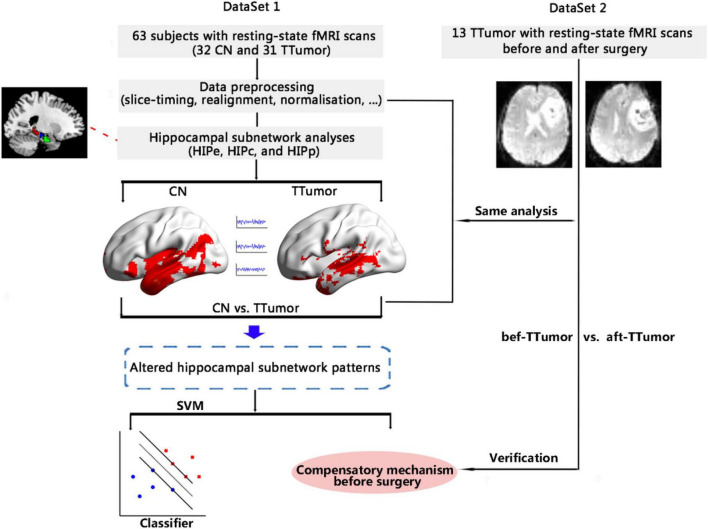
Schematic of the data analysis pipeline. Firstly, we measured functional connectivity (FC) within hippocampal subnetworks to identify any altered hippocampal subnetwork patterns in 32 CN and 31 TTumor subjects from the first cohort, as well as to identify preoperative compensatory brain areas. Secondly, we further applied a pattern classification approach (SVM) to evaluate whether altered hippocampal subnetwork patterns were able to distinguish TTumor from CN subjects. Finally, to test the stability of brain compensation mechanisms in preoperative TTumor glioma patients, we compared abnormal patterns of the hippocampal subnetwork before and after surgery in the second cohort. CN, healthy controls; TTumor, patients with temporal tumor (glioma); HIPe, hippocampal emotional region; HIPc, hippocampal cognitive region; HIPp, hippocampal perceptual region; fMRI, functional magnetic resonance imaging; SVM, support vector machine; bef-TTumor, patients with temporal tumor (glioma) before surgery; aft-TTumor, patients with temporal tumor (glioma) after surgery.

The objective of this study was to identify functional compensatory mechanisms of the hippocampal subnetwork in a cohort of TTumor patients through the use of a pattern classification approach (i.e., SVM). After identifying the compensatory mechanisms, we further investigated the stability of functional compensation in TTumor patients after undergoing surgical resection of the tumor in a separate cohort. Figure 1 shows the data analysis pipeline performed in this study.

## Materials and methods

### Subjects

Overall, this study recruited 44 patients with temporal tumors (TTumor) from two cohorts (31 patients from the first cohort and 13 patients from the second cohort). The entire cohort comprised of inpatients at the Department of Neurosurgery at the Affiliated Brain Hospital of Nanjing Medical University. In total, 32 healthy control (CN) volunteers were recruited through advertisements. Written informed consent was obtained from all participants. The study was approved by the Human Participants Ethics Committee of the Affiliated Brain Hospital of Nanjing Medical University, Nanjing, China.

Detailed inclusion and exclusion criteria are provided in our previously published studies (Hu et al., 2020; Liu et al., 2020a,c) and in Supplementary Methods 1.

### MRI data acquisition

The MRI data of TTumor patients were scanned between 2013 and 2022. The MRI images of TTumor patients were acquired before and 1 week after surgery using a 3.0 Tesla Verio Siemens scanner with an 8-channel head-coil that was provided by the department of radiology at the Affiliated Brain Hospital of Nanjing Medical University. The scanning parameters are provided in our previously published studies (Liu et al., 2019, 2020a,b) and in Supplementary Methods 2.

### Image preprocessing

The preprocessing steps in this study refer to previously published studies (Chen et al., 2020, 2022; Liu et al., 2020b) and are provided in SI Methods 3. MATLAB R2015b^1^ and SPM12^2^ were used to preprocess the MRI data. In brief, we conducted the following fMRI preprocessing steps: removing the first 10 images, slice-timing, head motion corrections, spatial normalization, spatial smoothing, nuisance covariates regression, and temporal filtering (0.01–0.1 Hz). Framewise displacement (FD) in the head motion parameter was not significantly different between the groups (*T* = −0.974, *p* = 0.334) (Power et al., 2012; Van Dijk et al., 2012). Furthermore, we calculated total intracranial volumes (TIV) using gray matter (GM), white matter (WM), and cerebrospinal fluid (CSF) partitions that were normalized and segmented using the Diffeomorphic Anatomical Registration Through Exponentiated Lie Algebra (DARTEL) method (Ashburner and Friston, 2009).

### Definition of hippocampal subregions

We defined our HIPsub based on recent studies by Robinson et al. (2015); Bai et al. (2019), and Chen et al. (2020, 2022). In brief, we defined the hippocampus as the following three subregions (Supplementary Figure 1): HIPe, HIPc, and HIPp. A detailed definition of the hippocampal subregion is provided in previous publications (Chen et al., 2020, 2022) and provided in Supplementary Methods 4.

### Hippocampal-subregion functional connectivity analyses

First, we created a group GM mask in the tumor-free region based on prior studies (Zhang et al., 2018; Di et al., 2022), and then the FC analysis of the HIPsub was conducted under the GM mask. The details regarding the construction of the GM mask in the tumor-free region are described in Supplementary Methods 5.

The steps of FC analysis of HIPsub are as follows. Firstly, the average time course for all voxels within HIPe, HIPc, and HIPp was extracted as the reference time course. Secondly, a voxel-wise cross-correlation analysis was conducted between the average time courses of all voxels within HIPe, HIPc, and HIPp and each voxel in the remainder of the whole brain within the group-specific GM mask. We defined the correlation coefficients obtained from this voxel-wise cross-correlation analysis as FC correlation coefficients. Finally, a Fisher’s z-transform analysis was conducted to improve the normality of the FC correlation coefficients.

### Pattern classification based on the altered functional connectivity within hippocampal subnetworks before surgery in the first cohort

In reference to prior studies (Chen et al., 2020, 2022), we applied an support vector machine (SVM) method in order to investigate the extent to which FC within the HIPsub network was able to distinguish TTumor from CN subjects. The LIBSVM software *(Software available at http://www.csie.ntu.edu.tw/∼cjlin/libsvm)* was then utilized to perform a linear SVM classifier. A leave-one-out cross-validation (LOOCV) strategy was then utilized to assess the generalization of this SVM classifier, and its sensitivity and specificity. We evaluated the power of this SVM classifier in order to discriminate TTumor from CN subjects with the use of receiver operating characteristic (ROC) curves. The area under the ROC (AUC) value was employed to evaluate the power.

### Changes of altered hippocampal-subregion networks related to temporal glioma tumor before and after surgery in in the second cohort

Based on the abnormal patterns that were present within the HIPsub networks before surgery (the first cohort), we compared the changes between these abnormal patterns before and after surgery in the second cohort using a paired *t*-test. Specifically, we tested the stability of brain compensation mechanisms among preoperative TTumor (glioma) patients.

### Statistical analysis

#### Demographics data

We conducted a two-sample independent *t*-test and chi-square tests (only applied in gender comparisons) to compare differences in demographic data, head movement parameters, and TIV between CN and TTumor groups. The level of statistical significance is set at *p* < 0.05.

#### Altered hippocampal-subregion network functional connectivity patterns related to temporal glioma tumor patients

In order to characterize the HIPsub network FC patterns at a group level, we carried out a random-effect analysis through the use of one-sample *t*-tests in the spatial maps of FC in CN and TTumor subjects with a stringent threshold of *p* < 0.05 using a permutation test with TFCE and the family-wise error (FWE) correction together with a cluster extent k > 100 voxels (2,700 mm^3^). *Then we created masks based on brain regions most robustly correlated with each HIPsub seed in TTumor and CN. FC data were extracted from the brain regions within these masks.*

*We conducted a general liner model (GLM) analysis to investigate differences in the FCs of HIPsub between the TTumor subjects and CN before surgery after controlling for age, sex, education, TIV, and mean FD (TFCE-FDR-corrected p* < *0.05 and cluster size* > *810 mm^3^).* Then we developed masks based on brain regions that demonstrated differences in the FCs of HIPsub in TTumor compared to CN. These masks were utilized for the analysis before vs. after surgery fMRI data.

## Results

### Demographic and neuropsychological characteristics

As shown in Table 1, no significant differences in age or gender were observed between the TTumor group and the CN group (all *p* > 0.05). TTumor group had lower education level than the CN subjects (*p* < 0.05).

**TABLE 1:** Demographics, head movement parameters, and total intracranial volumes of CN and TTumor subjects before surgery in the first cohort.

Items	CN (*n* = 32)	TTumor (*n* = 31)	*T*-value	*P*-value
Age (years)	58.97 (4.54)	53.58 (13.24)	2.175	0.034
Gender (M/F)	19/13	19/12	0.024	0.877
Education level (years)	11.97 (2.52)	11.93 (2.77)	5.483	<0.001
TIV	1634.21 (219.37)	1376.89 (543.14)	2.480	0.016
FD	0.10 (0.08)	0.13 (0.17)	−0.974	0.334

Values are expressed as the mean (standard deviation: SD). CN, healthy controls; TTumor, patients with temporal tumor (glioma); FD, framewise displacement; TIV, total intracranial volume.

### Identification of altered hippocampal-subregion networks related to temporal glioma tumor patients

As shown in Supplementary Figure 2, TTumor subjects displayed distinctly altered FC patterns of the three HIPsub networks (i.e., HIPe, HIPc, and HIPp networks) compared to CN (P_TFCE–FWE_ < 0.05, cluster size > 2,700 mm^3^).

**FIGURE 2:**
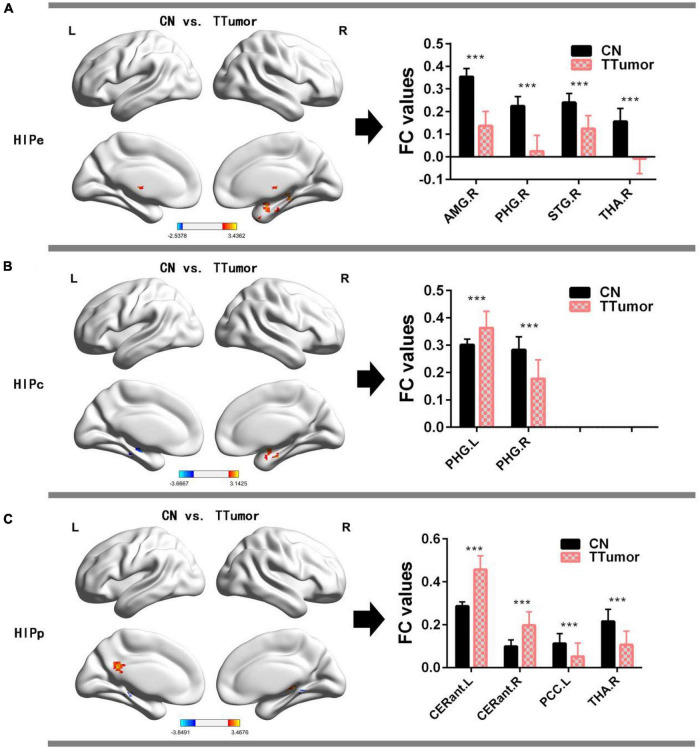
Comparison of FC within the hippocampal subnetworks between CN and TTumor subjects before surgery in the first cohort. **(A)** Altered regions of FC within the HIPe subnetwork in TTumor patients compared to CN. The bar chart on the right shows the quantitative comparison of FC within these altered regions; ***p < 0.001. **(B)** Altered regions of FC within the HIPc subnetwork in TTumor patients compared to CN. The bar chart on the right shows a quantitative comparison of FC within these altered regions; ***p < 0.001. **(C)** Altered regions of FC within the HIPp subnetwork in TTumor patients compared to CN. The bar chart on the right shows the quantitative comparison of FC within these altered regions; ***p < 0.001. CN, healthy controls; TTumor, patients with temporal tumor (glioma); HIPe, hippocampal emotional region; HIPc, hippocampal cognitive region; HIPp, hippocampal perceptual region; AMG.R, right amygdala; PHG.R, right parahippocampal gyrus; STG.R, right superior temporal gyrus; THA.R, right thalamus; PHG.L, left parahippocampal gyrus; CERant.L, left cerebellum anterior lobe; CERant.R, right cerebellum anterior lobe; PCC.L, left posterior cingulate cortex; L, left hemisphere; R, right hemisphere; FC, functional connectivity.

Figure 2A and Table 2 show that, in the HIPe network, compared to CN, TTumor patients had significantly decreased FC in the right amygdala, right parahippocampal gyrus, right superior temporal gyrus, and right thalamus. Additionally, Figure 2B and Table 2 depict that, in the HIPc network, compared to CN, TTumor patients showed significantly decreased FC in the right parahippocampal gyrus, but increased FC in the left parahippocampal gyrus. Figure 2C and Table 2 indicate that, in the HIPp network, compared to CN, TTumor patients revealed significantly decreased FC in the left posterior cingulate cortex and right thalamus, but increased FC in bilateral cerebellum anterior lobes. All the results were controlled for age, sex, education, TIV, and FD.

**TABLE 2:** Comparison of FC in the hippocampal subnetwork between CN and TTumor subjects before surgery in the first cohort.

Brain regions	L/R	BA	MNI			*T*-values	Cluster size (mm^3^)
			x	y	z		
**HIPe functional connectivity**							
Thalamus	R	−	12 −3 0			3.3216	1,134
Superior temporal gyrus	R	38	39 3 −27			3.1413	864
Parahippocampal gyrus	R	35	21 −30 −9			3.4362	1,134
Amygdala	R	28	21 −12 −30			3.1462	2,133
**HIPc functional connectivity**							
Parahippocampal gyrus	L	28	3 −15 −21			3.1425	2,376
Parahippocampal gyrus	R	36	−36 −18 −15			−3.6667	837
**HIPp functional connectivity**							
Thalamus	R	27	15 −30 0			3.4676	1,107
Cerebellum anterior lobe	L	–	−12 −33 −24			−3.8491	2,268
Cerebellum anterior lobe	R	30	15 −45 −9			−2.7717	918
Posterior cingulate cortex	L	23	−6 −48 24			3.1096	945

CN, healthy controls; TTumor, patients with temporal tumor (glioma); FC, functional connectivity; HIPe, hippocampal emotional region; HIPc, hippocampal cognitive region; HIPp, hippocampal perceptual region; MNI, Montreal neurological institute; L, left hemisphere; R, right hemisphere.

### Classification of temporal glioma tumor patients based on the altered hippocampal-subregion network connectivity

**FIGURE 3:**
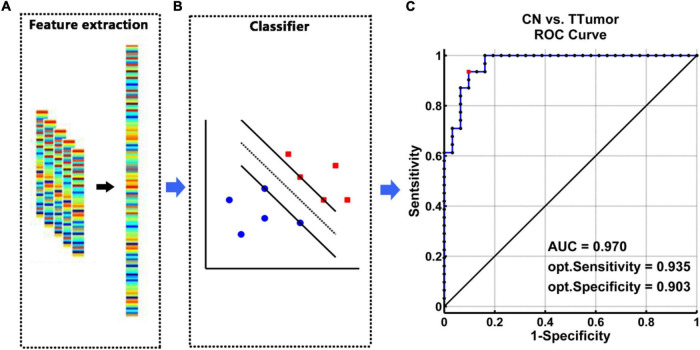
The classification power of an MRI-based “classifier” in distinguishing individuals with TTumor from CN before surgery in the first cohort. **(A)** We extracted features based on FC of the altered hippocampal subnetworks in order to train the “classifier” model. **(B)** The schematic diagram indicated a support vector machine “classifier,” based on altered functional characteristics within the HIPe, HIPc, and HIPp subnetworks. **(C)** The ROC curve showed the classification power of the MRI-based “classifier.” The values of AUC, sensitivity, and specificity are marked in the lower right of the figure. CN, healthy controls; TTumor, patients with temporal tumor (glioma); HIPe, hippocampal emotional region; HIPc, hippocampal cognitive region; HIPp, hippocampal perceptual region; AUC, area under the curve; FC, functional connectivity; opt, optimal.

The SVM classifier’s ROC curve revealed a high power to discriminate TTumor patients from CN on an individual subject basis, with an AUC of 97.0%, 93.5% sensitivity, and 90.3% specificity (Figure 3).

### Validation of stable compensatory mechanisms within hippocampal-subregion network in temporal glioma tumor patients

**FIGURE 4:**
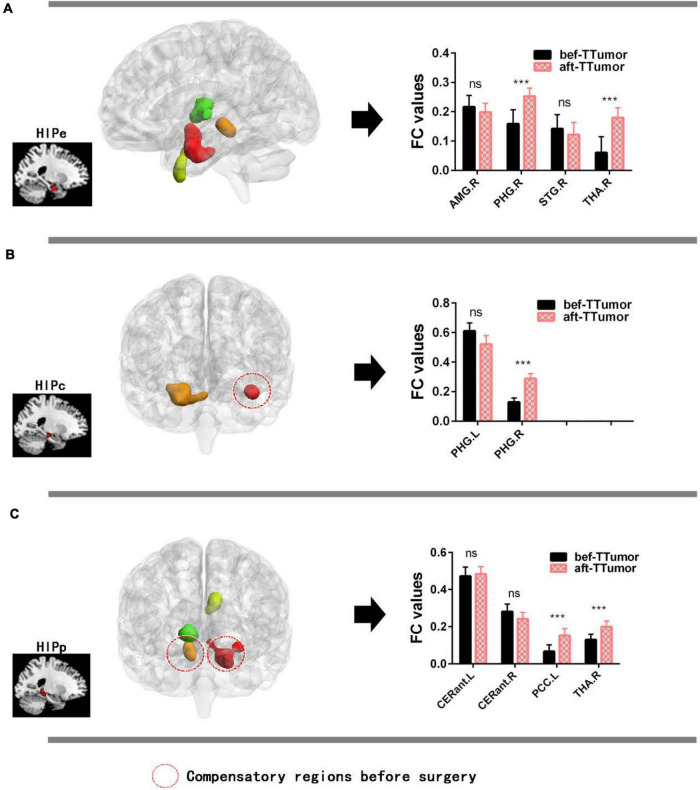
Changes on FC of altered hippocampal subnetworks in TTumor patients between before and after surgery in the second cohort controlling for age, sex, and education. **(A)** Bar chart showing quantitative effects on the HIPe subnetwork of TTumor patients after surgery after controlling for age, sex, and education. **(B)** Bar chart shows the quantitative effects on the HIPc subnetwork of TTumor patients after surgery after controlling for age, sex, and education. **(C)** Bar chart demonstrating the quantitative effects on HIPp subnetwork of TTumor patients post-surgery after controlling for age, sex, and education; ns, not significant. ***p < 0.001. Red circle indicates compensatory regions before surgery. CN, healthy controls; TTumor, patients with temporal tumor (glioma); HIPe, hippocampal emotional region; HIPc, hippocampal cognitive region; HIPp, hippocampal perceptual region; FC, functional connectivity; bef-TTumor, patients with temporal tumor (glioma) before surgery; aft-TTumor, patients with temporal tumor (glioma) after surgery; AMG.R, right amygdala; PHG.R, right parahippocampal gyrus; STG.R, right superior temporal gyrus; THA.R, right thalamus; PHG.L, left parahippocampal gyrus; CERant.L, left cerebellum anterior lobe; CERant.R, right cerebellum anterior lobe; PCC.L, left posterior cingulate cortex.

Figure 4A show that, in the HIPe network, compared to bef-TTumor, aft-TTumor patients demonstrated no differences in FC in the right amygdala and right superior temporal gyrus. They significantly decreased FC in the right parahippocampal gyrus and right thalamus. Figure 4B show that, in the HIPc network, compared to bef-TTumor, aft-TTumor patients showed no differences in FC in the left parahippocampal gyrus and significantly decreased FC in the right parahippocampal gyrus. Figure 4C show that, in the HIPp network, compared to bef-TTumor, aft-TTumor patients demonstrated no significant differences in FC in the bilateral cerebellum anterior lobe and significantly decreased FC in the left posterior cingulate cortex and right thalamus. All the results were controlled for age, sex, education, TIV, and FD.

## Discussion

To the best of our knowledge, particularly, the three most fascinating findings need to be emphasized. Firstly, tumor invasion of the temporal lobe resulted in abnormal HIPsub network connectivity and induced functional compensation of the HIPsub networks in the first cohort. Secondly, the altered HIPsub network connectivity patterns had a high power for discriminating TTumor patients from CN on an individual subject basis. *Finally, this study discovered that the FC of functional compensation regions in TTumor patients did not change before and after surgery in the second cohort.* Therefore, the unique contribution of this study was to identify functional compensation within the HIPsub network among patients with preoperative temporal glioma, which does not appear to be decompensated with tumor resection.

A highlight of this work is that when there is tumor invasion of the temporal lobe within HIPsub networks, TTumor patients demonstrated significantly different abnormal patterns in three HIPsub networks, which had the high power to discriminate TTumor patients from CN on an individual subject basis. *On the one hand, these results validate this view on functional heterogeneity and different FC patterns present in the hippocampal subregion* (Davachi, 2006; Robinson et al., 2015; Chen et al., 2016, 2020, 2022). On the other hand, this result also suggests that there is tumor invasion of the temporal lobe, which can affect brain function in a network form, and is consistent with prior studies (Bartolomei et al., 2006; He et al., 2007; Guggisberg et al., 2008; Alstott et al., 2009; Pravata et al., 2011; Liu et al., 2019, 2020b; Yuan et al., 2019). Furthermore, these results can enhance effective tools for early screening of tumors, and develop neurorehabilitation strategies for TTumor patients. *Interestingly, we discovered that FC decreased in brain regions of the functional hippocampal subnetwork caused by tumor invasion of the temporal lobe. However, it increased after tumor resection. Therefore, it is reasonable to speculate that the decrease in FC of these subhippocampal networks caused by tumor invasion of the temporal lobe may be a temporary brain functional abnormality that is caused by the tumor mass.* These results can further provide some theoretical basis for surgical planning of the resection range in clinical temporal lobe patients.

The unique contribution of this study is that tumor invasion of the temporal lobe was found toinduce functional compensation of the HIPsub networks in TTumor patients. This study found a spatial distance between these compensatory areas (i.e., left parahippocampal gyrus and bilateral cerebellum anterior lobes) and tumor areas. Indeed, many studies have reported that tumor invasion affects not only the tumor area but also the functional reorganization of the distal brain regions of the lesion (Thiel et al., 2001; Duffau, 2006; Desmurget et al., 2007). This may be because the effects of tumor invasion are network-based forms, rather than isolated brain regions (Vigneau et al., 2006; Xiang et al., 2010; Briganti et al., 2012; Liu et al., 2020b). These results suggest that tumor invasion of the temporal lobe helps induce functional compensation in the distal and proximal parts of the lesion in a network-based form. Furthermore, and most importantly, we discovered that functional compensation of the hippocampal subnetwork caused by tumor invasion of the temporal lobe in TTumor patients remained after tumor resection. This suggests that the compensatory mechanism formed during tumor invasion of the temporal lobe is stable, and not caused by tumor mass. Instead, it is a new mechanism to compensate for brain function, which will not be decompensated with tumor resection. One of the explanations may be that prolonged tumor invasion induces neuroplasticity of the central nervous system, which sustains cognitive function within the brain (Payne and Lomber, 2001; Voytek et al., 2010; Duffau, 2014). However, we need to be careful in coming to this conclusion as our observation point was only 1 week after surgery, and so we only confirmed the stability of this short-term compensation. Future long-term follow-up studies should be used to investigate and assess the stability of this compensation over time.

This study also has several limitations. Firstly, this study looked at only one point in time, 1 week after surgery, and this cannot verify long-term compensation for functional compensation. In the future, longitudinal studies are needed to confirm the stability and persistence of functional compensation within the HIPsub networks in TTumor patients. Secondly, the second cohort used in this study was a relatively small pre- and post-surgical sample. A larger sample study is required in the future to validate this compensatory mechanism.

## Conclusion

This study identifies that tumor invasion of the temporal lobe led to abnormal HIPsub network connectivity, and induced functional compensation of the HIPsub networks in TTumor patients, which can discriminate TTumor patients from CN on an individual subject basis. It has been suggested that the fine hippocampal subregion was more sensitive to reveal the functional compensation induced by tumor invasion of the temporal lobe. This study further validated the stability and persistence of this functional compensation in TTumor patients after surgical resection of the tumor. This study has significant implications that provide a novel avenue that avoids damage to the compensation of HIPsub networks, may protect against a brain function decline after surgery, and can improve the quality of life of patients.

## Data availability statement

The datasets presented in this study can be found in online repositories. The names of the repository/repositories and accession number(s) can be found in the article/Supplementary material.

## Ethics statement

The studies involving human participants were reviewed and approved by the Human Participants Ethics Committee of the Affiliated Brain Hospital of Nanjing Medical University. The patients/participants provided their written informed consent to participate in this study.

## Author contributions

YHZ and HX undertook the data analysis and wrote the manuscript. YL, KY, and YJZ acquired the data. HYL designed the study and provided infrastructure. YHZ, HX, and HYL supervised the data analysis. All authors contributed to and approved the final manuscript.

## Footnotes

1
http://www.mathworks.com/products/matlab/

2
https://www.fil.ion.ucl.ac.uk/spm/software/spm12/
