# Supplementary material for: Stable functional compensation within hippocampal-subregion networks in patients with temporal glioma before and after surgery
Source: Front Neurosci. 2022 Sep 1;16:991406. doi: 10.3389/fnins.2022.991406 (PMC9475067; doi:10.3389/fnins.2022.991406)
Supplement: Supplementary file 1 [file Data_Sheet_1.pdf]

Stable functional compensation within hippocampal-subregion networks in patients with temporal glioma before and after surgery

Supporting Information

SI methods

S.1 Inclusion and exclusion criteria
S.2 MRI scan parameters
S.3 Image preprocessing analysis
S.4 Definition of hippocampal subregions
S.5 Construction of group grey matter mask without tumor area

Supplementary figure legends

Figure S1 Schematic diagram of hippocampal subregions (sagittal views) in the left hemisphere.

Figure S2 Resting-state functional connectivity patterns of three hippocampal subnetworks within group maps in CN and TTumor subjects in DataSet 1.

Supporting Information

SI methods

S.1 Inclusion and exclusion criteria for patients

The inclusion criteria (Hu et al., 2020; Liu et al., 2020a; Liu et al., 2020c) for the patient groups were as follows: 1) tumor pathology was confirmed as glioma by surgery, 2) the extension of the tumor had not reached the central sulcus, 3) unilateral tumor invasion, 4) no brain injury, and 5) no history of biopsy, radiotherapy, or chemotherapy. The exclusion criteria (Hu et al., 2020; Liu et al., 2020a; Liu et al., 2020c) were: 1) multiple lesion foci, 2) history of substance abuse, and 3) magnetic resonance imaging (MRI) contraindications.

S.2 MRI scan parameters

The scan parameters were provided in our previously published studies (Liu et al., 2019; Liu et al., 2020a; Liu et al., 2020b).

T1-weighted MR images were obtained by a 3D magnetization-prepared rapid gradient echo (MPRAGE) with following parameters: repeat time (TR) = 1900 ms, echo time (TE) = 2.49 ms, time inversion (TI) = 900 ms, matrix = 256 x256, flip angle (FA) = 90°, thickness =1 mm, gap = 0.5 mm, slices = 176.

Resting-state functional images for patients were obtained in the same medical center with two set of scan parameters. The second set of scan parameters were used to obtain the MRI data for all CN subjects. Resting-state functional images including 140/240 volumes (for first set of parameter between 2013 and 2016 years/ second set of parameters between 2017 and 2019 years (Chen et al., 2019)) were obtained using a gradient-recalled echo-planar imaging (GRE-EPI) sequence, with repetition time (TR) = 2000 ms / 2000 ms, echo time (TE) = 30 ms / 30 ms, flip angle (FA) = 90° / 90°, acquisition matrix = 64 × 64 / 64 × 64, field of view (FOV) = 240 mm × 240 mm / 220 mm × 220 mm, thickness = 3.0 mm / 4.0 mm, gap = 4 mm / 0 mm, number of slices = 30 / 36, and voxel size = 3.75 × 3.75 × 4 mm3 / 3.4 × 3.4 × 4 mm3.

Note that the use of different parameters was due to the optimization and improvement of the imaging protocol of in our research team and was not related to the purposes of the study. Even if the parameters were homogeneous in the same scanner, parameters differences were taken into consideration in the GLM as a covariable of noninterest.

S.3 Image preprocessing analysis

The image processing procedure was as previously described (Chen et al., 2020; Chen et al., 2022; Liu et al., 2020b). We used MATLAB2016b (http://www.mathworks.com/products/matlab/) and DPABI to preprocess the MRI data (Yan et al., 2016). Firstly, we discarded the first ten images to minimize effects of scanner signal stabilization. Then we omitted scans with head motion exceeding 3mm or 3° of maximum rotation through the resting -state run. Then we calculated framewise displacement (FD) for all resting state volumes after realigning, slice timing correction, and co-registration (Power et al., 2012). All volumes with a FD greater than 0.2 mm were regressed out as nuisance covariates (Brady et al., 2019). Any scan with 50% of volumes removed

was discarded (Brady et al., 2019). Functional and structural images were co-registered. Structural images were then normalized and segmented into gray matter, white matter and cerebrospinal fluid signal (CSF) partitions using the DARTEL technique. The realigned fMRI data were normalized by using the EPI template into the standard MNI space and resampled to an isotropic voxel size of 3 mm, and then smoothed by a Gaussian kernel of 6 mm^3 full-width. Nuisance covarites regression including Friston 24-parameter model: 6 head motion parameters, 6 head motion parameters one time point before, and the 12 corresponding squared items (Friston et al., 1996), CSF, white matter, and the global signals as well as the linear trend were created and removed using partial regression with scrubbing (Power et al., 2014; Yan et al., 2013). After nuisance covariate regression, the resultant data were band pass filtered to select low frequency (0.01-0.1Hz) signals. Voxels within a group derived gray matter mask were used for further analyses.

S.4 Definition of hippocampal subregions

We defined our HIPsub in light of recent studies from Robinson et al. (Robinson et al., 2015), Bai et al.(Bai et al., 2019), and Chen (Chen et al., 2020; Chen et al., 2022). These studies used coactivation-based parcellation to reveal a subspecialization in the hippocampus. Their findings showed that the right hippocampal segmentation is ambiguous. Therefore, we selected only the left hippocampal subregions as regions of interest (ROI). Based on the recent study published by Chen and colleagues (Chen et al., 2020; Chen et al., 2022). The left hippocampus was defined as three subregions, which were appropriately located at the anterior involving in emotional processes (HIPe), middle involving in cognitive processes (HIPc), and posterior involving in perceptual function (HIPp). It is worth noting that the overlapping areas between adjacent clusters were removed as presented by Chen and colleagues (Chen et al., 2020; Chen et al., 2022), which may affect the subsequent analysis. The non-overlapping clusters were selected as ROIs for further analysis (Fig. S1).

S.5 Construction of group GM mask in the tumor-free area

Referring to previous studies (Di et al., 2022; Zhang et al., 2018), two senior neurosurgeons used MRIcroN software to manually trace tumors on individual 3D structural images: T1 enhancement images for gliomas with enhancement, and T2-flair images coregistered to T1 weighted images for gliomas without enhancement. The third senior neurosurgeon confirmed the accuracy of the manual tracing.Then a tumor mask for each patient was created after manually tracing the tumor. This tumor mask is spatially normalized to the standard MNI template, and we binarized and tacked all patients' tumor masks together to construct a tumor overlapping region. We further calculated the intersection of this tumor overlapping region and the gray matter mask (obtained by thresholding the gray matter probability map in SPM12 with probability larger than 0.2). Then we use this GM mask to reduce the intersection mask to obtain the final patients' group GM mask.

Supplementary figures and legends

Figure S1.

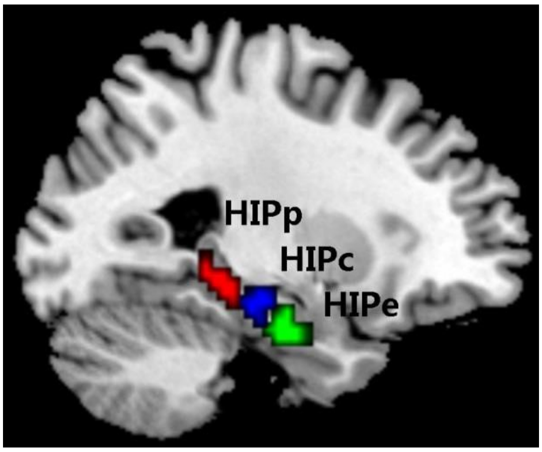

Fig. S1. Schematic diagram of hippocampal subregions (sagittal views) in the left hemisphere. Hippocampal subregions were referred to recent studies published by Robinson et al. (Robinson et al., 2015) and Chen (Chen et al., 2020; Chen et al., 2022), who used coactivation-based parcellation to reveal a subspecialization in the hippocampus by a data-driven method. Hippocampal subregions included HIPe (green), HIPc (blue), and HIPp (red). Noteworthy, we removed the overlapping areas between adjacent clusters as referred to Chen (Chen et al., 2020; Chen et al., 2022), which may affect the subsequent analysis. Abbreviations: HIPe, hippocampal emotional region; HIPc, hippocampal cognitive region; HIPp, hippocampal perceptual region.

Figure S2

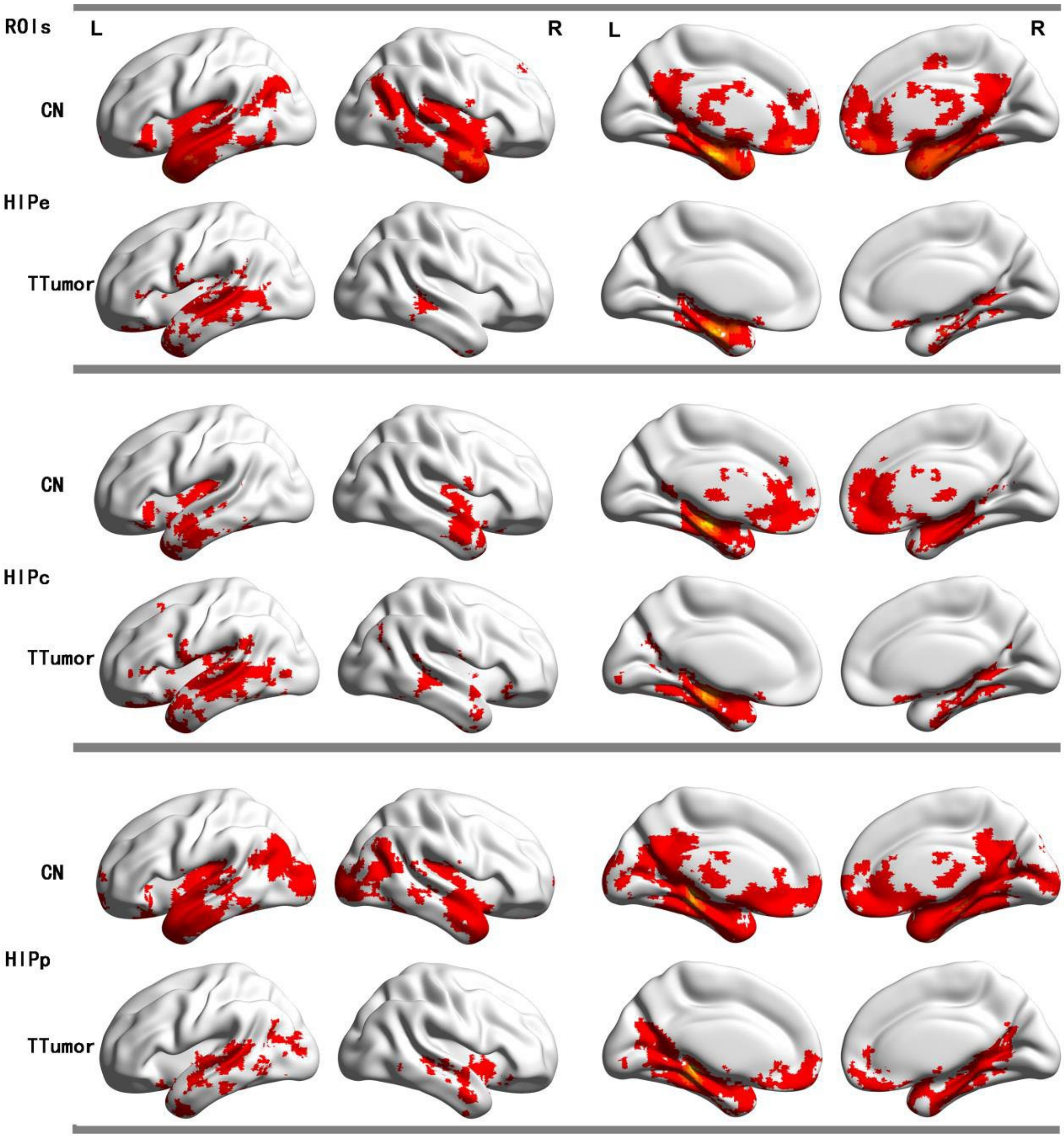

Figure S2. Resting-state functional connectivity patterns of three hippocampal subnetworks within group maps in CN and TTumor subjects in DataSet 1.

Abbreviations: CN, healthy controls; TTumor, patIents with temporal tumor (glioma); HIPe, hippocampal emotional region; HIPc, hippocampal cognitive region; HIPp, hippocampal perceptual region; ROIs, regions of Interest; L, left hemisphere; R, right hemisphere.

.

References

Bai, T., Wei, Q., Xie, W., Wang, A., Wang, J., Ji, G.J., Wang, K., Tian, Y., 2019. Hippocampal-subregion functional alterations associated with antidepressant effects and cognitive impairments of electroconvulsive therapy. Psychol Med. 49, 1357-1364.

Brady, R.O., Jr., Gonsalvez, I., Lee, I., Ongur, D., Seidman, L.J., Schmahmann, J.D., Eack, S.M., Keshavan, M.S., Pascual-Leone, A., Halko, M.A., 2019. Cerebellar-Prefrontal Network Connectivity and Negative Symptoms in Schizophrenia. Am J Psychiatry. appiajp201818040429.

Chen, J., Chen, G., Shu, H., Chen, G., Ward, B.D., Wang, Z., Liu, D., Antuono, P.G., Li, S.J., Zhang, Z., Alzheimer's Disease Neuroimaging, I., 2019. Predicting progression from mild cognitive impairment to Alzheimer's disease on an individual subject basis by applying the CARE index across different independent cohorts. Aging (Albany NY). 11, 2185-2201.

Chen, J., Ma, N., Hu, G., Nousayhah, A., Xue, C., Qi, W., Xu, W., Chen, S., Rao, J., Liu, W., Zhang, F., Zhang, X., 2020. rTMS modulates precuneus-hippocampal subregion circuit in patients with subjective cognitive decline. Aging (Albany NY). 12.

Chen, J., Chen, R., Xue, C., Qi, W., Hu, G., Xu, W., Chen, S., Rao, J., Zhang, F., Zhang, X., 2022. Hippocampal-Subregion Mechanisms of Repetitive Transcranial Magnetic Stimulation Causally Associated with Amelioration of Episodic Memory in Amnestic Mild Cognitive Impairment. J Alzheimers Dis. 85, 1329-1342.

Di, G., Tan, M., Xu, R., Zhou, W., Duan, K., Hu, Z., Cao, X., Zhang, H., Jiang, X., 2022. Altered Structural and Functional Patterns Within Executive Control Network Distinguish Frontal Glioma-Related Epilepsy. Front Neurosci. 16, 916771.

Friston, K.J., Williams, S., Howard, R., Frackowiak, R.S., Turner, R., 1996. Movement-related effects in fMRI time-series. Magn Reson Med. 35, 346-55.

Hu, G., Hu, X., Yang, K., Liu, D., Xue, C., Liu, Y., Xiao, C., Zou, Y., Liu, H., Chen, J., 2020. Restructuring of contralateral gray matter volume associated with cognition in patients with unilateral temporal lobe glioma before and after surgery. Hum Brain Mapp. 41, 1786-1796.

Liu, D., Hu, X., Liu, Y., Yang, K., Xiao, C., Hu, J., Li, Z., Zou, Y., Chen, J., Liu, H., 2019. Potential Intra- or Cross-Network Functional Reorganization of the Triple Unifying Networks in Patients with Frontal Glioma. World Neurosurg.

Liu, D., Chen, J., Hu, X., Hu, G., Liu, Y., Yang, K., Xiao, C., Zou, Y., Liu, H., 2020a. Contralesional homotopic functional plasticity in patients with temporal glioma. J Neurosurg. 1-9.

Liu, Y., Hu, G., Yu, Y., Jiang, Z., Yang, K., Hu, X., Li, Z., Liu, D., Zou, Y., Liu, H., Chen, J., 2020b. Structural and Functional Reorganization Within Cognitive Control Network Associated With Protection of Executive Function in Patients With Unilateral Frontal Gliomas. Front Oncol. 10, 794.

Liu, Y., Yang, K., Hu, X., Xiao, C., Rao, J., Li, Z., Liu, D., Zou, Y., Chen, J., Liu, H., 2020c. Altered Rich-Club Organization and Regional Topology Are Associated With Cognitive Decline in Patients With Frontal and Temporal Gliomas. Front Hum Neurosci. 14, 23.

Power, J.D., Barnes, K.A., Snyder, A.Z., Schlaggar, B.L., Petersen, S.E., 2012. Spurious but systematic correlations in functional connectivity MRI networks arise from subject motion. Neuroimage. 59**,** 2142-54.

Power, J.D., Mitra, A., Laumann, T.O., Snyder, A.Z., Schlaggar, B.L., Petersen, S.E., 2014. Methods to detect, characterize, and remove motion artifact in resting state fMRI. Neuroimage. 84**,** 320-41.

Robinson, J.L., Barron, D.S., Kirby, L.A., Bottenhorn, K.L., Hill, A.C., Murphy, J.E., Katz, J.S., Salibi, N., Eickhoff, S.B., Fox, P.T., 2015. Neurofunctional topography of the human hippocampus. Hum Brain Mapp. 36**,** 5018-37.

Yan, C.G., Craddock, R.C., Zuo, X.N., Zang, Y.F., Milham, M.P., 2013. Standardizing the intrinsic brain: towards robust measurement of inter-individual variation in 1000 functional connectomes. Neuroimage. 80**,** 246-62.

Yan, C.G., Wang, X.D., Zuo, X.N., Zang, Y.F., 2016. DPABI: Data Processing & Analysis for (Resting-State) Brain Imaging. Neuroinformatics. 14**,** 339-51.

Zhang, N., Xia, M., Qiu, T., Wang, X., Lin, C.P., Guo, Q., Lu, J., Wu, Q., Zhuang, D., Yu, Z., Gong, F., Farrukh Hameed, N.U., He, Y., Wu, J., Zhou, L., 2018. Reorganization of cerebro-cerebellar circuit in patients with left hemispheric gliomas involving language network: A combined structural and resting-state functional MRI study. Hum Brain Mapp. 39**,** 4802-4819.
